# High-resolution food webs based on nitrogen isotopic composition of amino acids

**DOI:** 10.1002/ece3.1103

**Published:** 2014-05-17

**Authors:** Yoshito Chikaraishi, Shawn A Steffan, Nanako O Ogawa, Naoto F Ishikawa, Yoko Sasaki, Masashi Tsuchiya, Naohiko Ohkouchi

**Affiliations:** 1 Japan Agency for Marine-Earth Science and Technology2-15 Natsushima-cho, Yokosuka, 237-0061, Japan; 2 USDA-ARS Vegetable Crops Research Unit, 1630 Linden Dr., Department of Entomology, University of WisconsinMadison, WI, 53706, USA

**Keywords:** Carnivores, compound-specific isotope analysis, ecosystem, herbivores, omnivores, predators, primary producers, trophic position

## Abstract

Food webs are known to have myriad trophic links between resource and consumer species. While herbivores have well-understood trophic tendencies, the difficulties associated with characterizing the trophic positions of higher-order consumers have remained a major problem in food web ecology. To better understand trophic linkages in food webs, analysis of the stable nitrogen isotopic composition of amino acids has been introduced as a potential means of providing accurate trophic position estimates. In the present study, we employ this method to estimate the trophic positions of 200 free-roaming organisms, representing 39 species in coastal marine (a stony shore) and 38 species in terrestrial (a fruit farm) environments. Based on the trophic positions from the isotopic composition of amino acids, we are able to resolve the trophic structure of these complex food webs. Our approach reveals a high degree of trophic omnivory (i.e., noninteger trophic positions) among carnivorous species such as marine fish and terrestrial hornets.This information not only clarifies the trophic tendencies of species within their respective communities, but also suggests that trophic omnivory may be common in these webs.

## Introduction

Recent studies have emphasized the importance of functional diversity in the provision of ecosystem services (Duffy et al. 2007; Griffin et al. 2008). Assessing the trophic niche of a species, however, has remained difficult, partly because there is little consensus as to appropriate metrics (Chase and Leibold 2003), and partly because there are so few empirical approaches that permit accurate and precise measurements of the feeding histories of animals (Chikaraishi et al. 2011; Steffan et al. 2013). This is particularly true for omnivores and higher-order consumers, where such groups are often left as large, undivided units rather than parsed into smaller trophic subsets (e.g., Polis and Strong 1996; Sih et al. 1998).

Evidence for the importance of omnivory in food webs has long been reported (e.g., Darnell 1961; Polis 1991; Coll and Guershon 2002; Bruno and O'Connor 2005). Indeed, multichannel omnivory has been postulated as a dominant feature of carnivore communities (Polis 1991; Polis and Strong 1996), with much subsequent support of this pattern (Rosenheim 1998; Coll and Guershon 2002; Williams and Martinez 2004; Finke and Denno 2005). Recent work suggests that species feeding above the level of strict herbivory are often a “tangled web” of trophic omnivores (Thompson et al. 2007), feeding opportunistically yet often expressing distinct trophic tendencies (Minagawa and Wada 1984; Power et al. 1985; Vander Zanden and Rasmussen 2001; Post 2002; Williams and Martinez 2004). These tendencies often exhibit characteristic variability (Jaksić and Delibes 1987; Bearhop et al. 2004), and such variation represents the “trophic spectrum” of a species (Polis and Strong 1996). Understanding trophic spectra may be critical to assessing the functional diversity of ecosystems, not only because the spectra provide information as to the variability, or range of trophic roles played by consumer species, but also because they indicate the central tendency of these species. Thus, measuring trophic spectra empirically should help tease apart the tangle of higher-order consumption by effectively characterizing the trophic niches of omnivores and carnivores.

**Figure 1:**
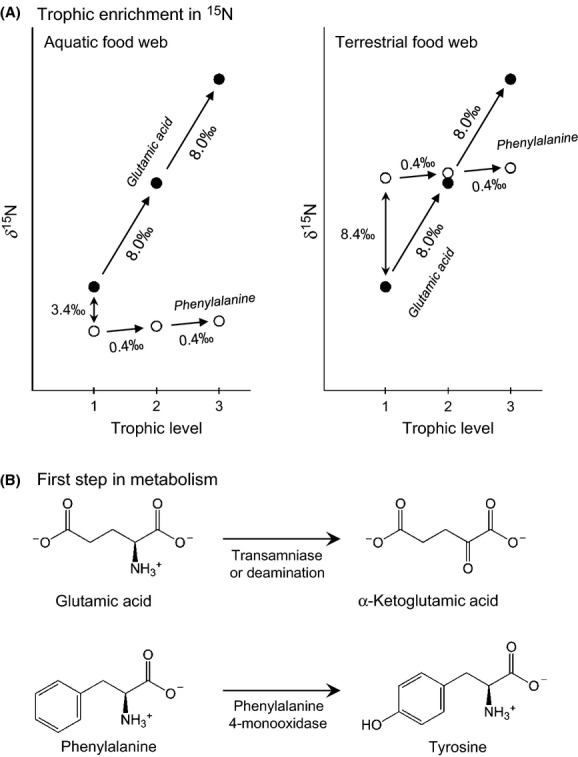
(A) Schematic illustration of the relationship between *δ*^15^N values of amino acids (glutamic acid and phenylalanine) and trophic level in food webs (after Chikaraishi et al. 2007, 2009), and (B) initial steps of the dominant metabolism for glutamic acid and phenylalanine in animals.

Knowledge of the trophic position (TP) of organisms in food webs allows ecologists to track biomass flow, apportionment among trophic groups, and the trophic compositions of communities (e.g., Pimm 1991; Post 2002; Williams and Martinez 2004). Analysis of the stable nitrogen isotopic composition (*δ*^15^N) of amino acids represents a relatively new method that has been shown to provide accurate and precise estimates of the trophic position of organisms in aquatic and terrestrial systems (e.g., McClelland and Montoya 2002; McCarthy et al. 2007; Popp et al. 2007; Chikaraishi et al. 2009; Steffan et al. 2013). This approach is based on contrasting isotopic fractionation during metabolic processes between “trophic” and “source” amino acids (TrAAs and SrcAAs, respectively). For example, glutamic acid, a representative TrAA, shows significant ^15^N-enrichment (8.0‰ on average) during the transfer of biomass from one trophic level to another because its metabolism starts with transamination/deamination, which always cleaves carbon–nitrogen bonds (Fig. 1). Conversely, phenylalanine, a representative SrcAA, shows little ^15^N-enrichment (+0.4‰ on average) because its metabolism begins with the conversion of phenylalanine into tyrosine, which neither forms nor cleaves carbon–nitrogen bonds (Fig. 1). Thus, given the minimal enrichment of SrcAAs with each trophic transfer, the isotopic composition of SrcAAs in consumers represents the weighted average of all the resource species at the base of the food web. As an organism feeds higher in its food web, the *δ*^15^N value of TrAAs elevates predictably, while SrcAAs remains relatively static. A comparison of the isotopic composition between these two types of amino acids in any organism corresponds closely to the feeding position held by that organism within its food web (Steffan et al. 2013). In previous studies involving natural and laboratory-reared organisms, we established a general equation for the empirical measurement of an organism's trophic position:


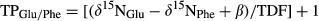
(1)

where the *β* represents the isotopic difference between glutamic acid (*δ*^15^N_Glu_) and phenylalanine (*δ*^15^N_Phe_) in primary producers (−3.4 ± 0.9‰ for aquatic cyanobacteria and algae, +8.4 ± 1.6‰ for terrestrial C_3_ plants, −0.4 ± 1.7‰ for terrestrial C_4_ plants), and the TDF represents trophic discrimination factor (7.6 ± 1.2‰ = *Δ*^15^N_Glu_ − *Δ*^15^N_Phe_) at each shift of trophic level (Chikaraishi et al. 2010). Also, several previous studies used or suggested an alternative equation using a combination of all available isotopic composition (*δ*^15^N) of TrAAs and SrcAAs:


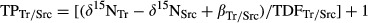
(2)

where the *β*_Tr/Src_ represents the isotopic difference between the weighted mean isotopic composition of TrAAs (*δ*^15^N_Tr_) and SrcAAs (*δ*^15^N_Src_) in primary producers, and the TDF_Tr/Src_ represents the TDF between TrAAs and SrcAAs (i.e., = *Δ*^15^N_Tr_ − *Δ*^15^N_Src_) (e.g., Sherwood et al. 2011; Décima et al. 2013; Vander Zanden et al. 2013).

Using this method, the TP value is calculated as a linear function of the difference in the *δ*^15^N values of amino acids from the organism of interest (Chikaraishi et al. 2009; Steffan et al. 2013). As a result, the TP calculation accounts for the natural background variation in the nitrogen isotopic composition. In fact, previous studies reported that the standard deviation (1*σ*) of the accuracy of TP_Glu/Phe_ value (= [actual TP] − [TP_Glu/Phe_]) was only 0.12 unit among aquatic species and 0.17 unit among terrestrial organisms, while the variability in the isotopic composition at the base of the food webs ranging up to ∼15‰ (Chikaraishi et al. 2009, 2011). The potential uncertainty in the TP_Glu/Phe_ value calculated by taking into account the propagation of uncertainty on each factor in Eq. (1) is also only 0.23–0.24, 0.26–0.30, and 0.36–0.43 units for primary producers, primary consumers, and secondary consumers, respectively, in the terrestrial food web (Chikaraishi et al. 2011). This is a key advantage of this method and stands in contrast to traditional trophic position estimation techniques that rely on the nitrogen isotopic composition of bulk tissue samples (e.g., DeNiro and Epstein 1981; Minagawa and Wada 1984). The traditional bulk-analysis method is highly sensitive to background isotopic variation between the basal resources of a food web (e.g., Cabana and Rasmussen 1996; Vander Zanden et al. 1997; Vander Zanden and Rasmussen 1999; Post 2002). Another advantage of the amino acid approach is that it permits analyses of exceedingly small specimens (2 nmol for each amino acid, Chikaraishi et al. 2009), which allows researchers to assess the trophic functions of innumerable micro- and meso-fauna. Finally, the amino acid method is applicable to not only modern samples but also formalin-fixed and fossil (e.g., bone collagen) samples (Naito et al. 2010, 2013; Styring et al. 2010, 2012; Ogawa et al. 2013). Because of these advantages, the estimation of trophic position based on the isotopic composition of amino acids has been used with various organisms in recent ecological studies (e.g., McClelland et al. 2003; Hannides et al. 2009; Lorrain et al. 2009; Bloonfield et al. 2011; Dale et al. 2011; Sherwood et al. 2011; Maeda et al. 2012; Miller et al. 2012; Germain et al. 2013; Ruiz-Cooley et al. 2013; Vander Zanden et al. 2013).

However, the validity of this estimate is dependent on the consistency of both *β* and TDF values. Recent studies reported potentially little or substantial variation in the *β* value for cyanobacteria and algae (McCarthy et al. 2013), seagrass (Vander Zanden et al. 2013), and terrestrial C_3_ plants (Steffan et al. 2013). It was confirmed that the TDF value does not scale among trophic levels 1–4 in multiple controlled-feeding experiments and for trophic levels 1–5 in a natural food chain using terrestrial arthropod species (Steffan et al. 2013); however, the universality of the TDF has been questioned for several species, including penguins (Lorrain et al. 2009), elasmobranches (Dale et al. 2011), jumbo squids (Ruiz-Cooley et al. 2013), and harbor seals (Germain et al. 2013). In these species, small TDF values (3–5‰) were consistent with traditional biological observations such as stomach content analysis.

However, these biological observations did not involve empirical measurement of prey trophic position, and even if the prey trophic positions had been assayed, they would only have represented a snap-shot of the animal's feeding history. Thus, without lifelong measurements of prey trophic position, there is little basis to assert that TFDs of free-roaming marine species may be significantly different from the TDFs reported in controlled-feeding studies. Altogether, these results indicate that the *β* and TDF parameters are quite useful but would benefit from further refinement, particularly via controlled-feeding experiments involving various species, conditions, and positions within trophic hierarchies.

In the present study, we apply this method to investigations of selected flora and fauna in coastal marine (a stony shore) and terrestrial (a fruit farm) ecosystems in Japan. We aggregate data reported in previous studies (Chikaraishi et al. 2009, 2010, 2011) and report the TP_Glu/Phe_ values of a total of 200 samples represented by 100 samples from 39 species in the coastal and 100 samples from 38 species in the terrestrial food webs (Table 1). Based on the observed TP_Glu/Phe_ values, we illuminate elements of the food web structure in these ecosystems and further evaluate this new method of food web analysis.

**Table 1:** Coastal marine and terrestrial organisms included in the present study

	Number of samples				Number of samples		
			
Sample	Ref 1^1^	Ref 2^1^	This study	Sample	Ref 2^1^	Ref 3^1^	This study
Marine costal (stony shore)				Terrestrial (fruit farm)			
Macroalgae (Brown algae)				Plant			
*Undaria pinnatifida*	1	1		*Brassica oleracea*	3		
*Sargassum filicinum*	2			*Daucus carota*		1	
*Ecklonia cava*			1	*Castanea crenata*	2		1
*Eisenia bicyclis*			1	*Citrus unshiu*		1	
Macroalgae (Red algae)				*Cucurbita moschata*			1
*Binghamia californica*	1			*Diospyros kaki Thunberg*			1
*Gelidium japonicum*	2		3	*Prunus avium*			1
Gastropod				*Raphanus sativus*		1	
*Batillus cornutus*	1		1	*Solanum lycopersicum*		1	
*Haliotis discus*	1		1	*Solanum melongena*		1	
*Omphalius pfeifferi*	1		4	*Solanum tuberosum*		1	
Echinoid				Aphid			
*Anthocidaris crassispina*			1	*Aphidoidea* sp.		1	
*Hemicentrotus pulcherrimus*			1	Butterfly			
Oyster				*Hestina assimilis*			1
*Crassostrea* sp.			1	*Papilio machaon*			1
Crustacea				*Papilio protenor*			1
*Pachygrapsus crassipes*	1		1	*Pieris rapae* (caterpillar)	2		2
*Pagurus filholi*			1	*Pieris rapae*			2
*Panulirus japonicus*			5	Bee			
*Plagusia dentipes*	1			*Apis mellifera*		3	
*Percnon planissimum*	1			*Bombus diversus diversus*		1	1
*Pugettia quadridens*			1	*Xylocopa appendiculata circumvolans*		1	1
*Thalamita pelsarti Montgomery*			1	Katydid			
Fish				*Gampsocleis mikado*			1
*Acanthopagrus schlegeli*	1			*Holochlora japonica*			1
*Apogon semilineatus*			11	Paper wasp			
*Canthigaster rivulata*			1	*Polistes japonicus japonicus*		6	
*Ditrema temmincki temmincki*			1	*Polistes jokahamae jokahamae*			3
*Girella punctata*	1		14	*Polistes mandarinus*			1
*Gymnothorax kidako*			3	*Polistes rothneyi iwatai*		14	
*Goniistius zonatus*			1	*Parapolybia indica*		9	
*Halichoeres poecilopterus*			3	Ant			
*Lutjanus stellatus*			1	*Formica japonica*			1
*Microcanthus strigatus*			3	Ladybug			
*Oplegnathus fasciatus*			2	*Coccinella septempunctata*			2
*Oplegnathus punctatus*			1	*Harmonia axyridis*		3	4
*Parapristipoma trilineatum*			5	*Illeis koebelei*			5
*Pseudoblennius percoides*			1	*Menochilus sexmaculatus*			2
*Pseudolabrus siebold*			5	Mantis			
*Pteragogus flagellifer*			1	*Tenodera aridifolia*			1
*Sebastes inermis*			2	Hornet			
*Sebastiscus marmoratus*			5	*Vespa analis fabriciusi*			7
*Takifugu niphobles*			1	*Vespa ducalis pulchra*		3	
Octopus				*Vespa mandarinia japonica*		1	2
*Octopus vulgaris*			1	*Vespa simillima xanthoptera*		1	
				*Vespula flaviceps lewisii*		1	

1 Ref 1: Chikaraishi et al. 2009; Ref 2: Chikaraishi et al. 2010a; Ref 3: Chikaraishi et al. 2011.

## Materials and Methods

All of the marine and terrestrial samples were collected in 2001–2013 from a stony shore and a farm in Yugawara (35°08′N, 139°07′E), Japan, respectively. The stony shoreline surveyed represented ∼0.2 hectares and ranged in depth from 0 to 5 m, where brown and red macroalgae are dominant primary producers but seagrass is absent. The farm was also approximately 0.2 hectares with cultivation of fruits and vegetables, all of which were C_3_ plants. Green leaves and/or nuts were collected for higher plants, and whole samples of 1–15 individuals within a single stage were collected for the other species. The collected samples were cleaned with distilled water to remove surface contaminants and stored at −20°C. For most terrestrial species and marine macroalgae, whole-organism samples were prepared for isotopic analyses. For the remaining marine specimens, small samples of muscle tissue were taken. Shell samples were taken from several gastropod and lobster specimens, and scales were dissected from most of the fish species (Appendices A1 and A2). There was no substantial effect on the trophic position estimates among these different tissue types within a single animal specimen (e.g., Chikaraishi et al. 2010, 2011; Ogawa et al. 2013). The bulk-carbon and bulk-nitrogen isotopic compositions of representative samples (40 coastal marine and 69 terrestrial samples, Appendices A1 and A2) were determined using a Flash EA (EA1112) instrument coupled to a Delta^plus^XP IRMS instrument with a ConFlo III interface (Thermo Fisher Scientific, Bremen, Germany). Carbon and nitrogen isotopic compositions are reported in the standard delta (*δ*) notation relative to the Vienna Peedee Belemnite (VPDB) and to atmospheric nitrogen (AIR), respectively.

The nitrogen isotopic composition of amino acids was determined by gas chromatography/combustion/isotope ratio mass spectrometry (GC/C/IRMS) after HCl hydrolysis and *N*-pivaloyl/isopropyl (Pv/iPr) derivatization, according to the procedure in Chikaraishi et al. (2009) (which are described in greater detail at http://www.jamstec.go.jp/biogeos/j/elhrp/biogeochem/download_e.html). In brief, samples were hydrolyzed using 12 Mol/L HCl at 110°C. The hydrolysate was washed with *n*-hexane/dichloromethane (3/2, v/v) to remove hydrophobic constituents. Then, derivatizations were performed sequentially with thionyl chloride/2-propanol (1/4) and pivaloyl chloride/dichloromethane (1/4). The Pv/iPr derivatives of amino acids were extracted with *n*-hexane/dichloromethane (3/2, v/v). The nitrogen isotopic composition of amino acids was determined by GC/C/IRMS using a 6890N GC (Agilent Technologies, Palo Alto, CA) instrument coupled to a Delta^plus^XP IRMS instrument via a GC-C/TC III interface (Thermo Fisher Scientific, Bremen, Germany). To assess the reproducibility of the isotope measurement and obtain the amino acid isotopic composition, reference mixtures of nine amino acids (alanine, glycine, leucine, norleucine, aspartic acid, methionine, glutamic acid, phenylalanine, and hydroxyproline) with known *δ*^15^N values (ranging from −25.9‰ to +45.6‰, Indiana University, SI science co.) were analyzed after every four to six samples runs, and three pulses of reference N_2_ gas were discharged into the IRMS instrument at the beginning and end of each chromatography run for both reference mixtures and samples. The isotopic composition of amino acids in samples was expressed relative to atmospheric nitrogen (AIR) on scales normalized to known *δ*^15^N values of the reference amino acids. The accuracy and precision for the reference mixtures were always 0.0‰ (mean of *Δ*) and 0.4–0.7‰ (mean of 1*σ*) for sample sizes of ≥1.0 nmol N, respectively.

The *δ*^15^N values were determined for the following 10 amino acids: alanine, glycine, valine, leucine, isoleucine, proline, serine, methionine, glutamic acid, and phenylalanine (Appendices A1 and A2). These amino acids were chosen because their peaks were always well separated with baseline resolution in the chromatogram (Chikaraishi et al. 2009). Also, it should be noted that glutamine was quantitatively converted to glutamic acid during acid hydrolysis; as a result, the *α*-amino group of glutamine contributed to the *δ*^15^N value calculated for glutamic acid.

The TP_Glu/Phe_ value (and its potential uncertainty calculated by taking into account the propagation of uncertainty on each factor in the Eq. (1)) was calculated from the observed *δ*^15^N values (as 1*σ* = 0.5‰) of glutamic acid and phenylalanine in the organisms of interest, using eq. (1) with the *β* value of −3.4 ± 0.9‰ for coastal marine and +8.4 ± 1.6‰ for terrestrial samples, and with the TDF value of 7.6 ± 1.2‰ for both ecosystems, according to Chikaraishi et al. (2009, 2010, 2011). The TP_Tr/Scr_ values were not calculated, because we did not measure the *δ*^15^N values of lysine and tyrosine for all investigated samples and of serine for approximately a half of samples.

## Results and Discussion

### *δ*^13^C and *δ*^15^N values of bulk samples

**Figure 2:**
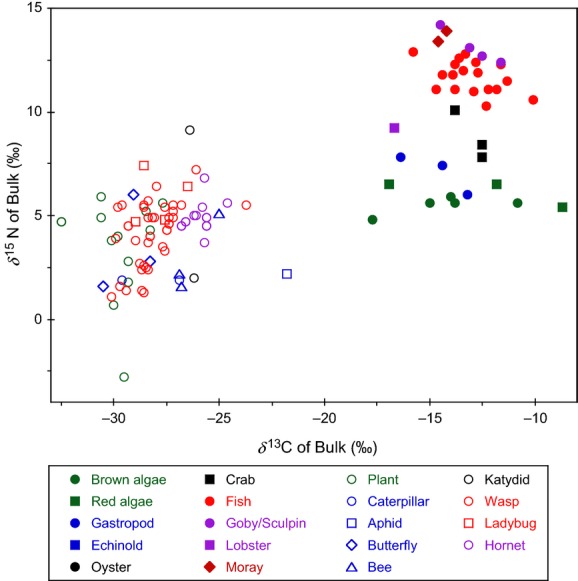
*δ*^13^C and *δ*^15^N values of bulk samples.

Carbon and nitrogen isotopic compositions of bulk samples ranged from −17.7‰ to −8.7‰ and from +4.8‰ to +14.2‰, respectively, within the coastal marine system (Appendix A1). In the terrestrial system, respective carbon and nitrogen isotopic compositions ranged from −32.5‰ to −21.8‰ and from −2.8‰ to +9.1‰ (Appendix A2). These two ecosystems are readily distinguished in the *δ*^13^C-*δ*^15^N cross-plot of the organisms, mainly because of disparity in the *δ*^13^C value of the food web resource between coastal marine and terrestrial systems (Fig. 2).

In the present study, the nitrogen isotopic composition ranges from +4.8‰ to +7.8‰ for marine algae and from −2.8‰ to +5.9‰ for the terrestrial plants. This heterogeneity in the isotopic composition of basal resources, particularly in the terrestrial system, was relatively large up to 2.6 times as large as the discrimination factor (i.e., 3.4‰; Minagawa and Wada 1984), which is used to estimate the trophic position based on bulk isotopic composition.

### Precision of TP_Glu/Phe_ for multiple sample analysis

**Figure 3:**
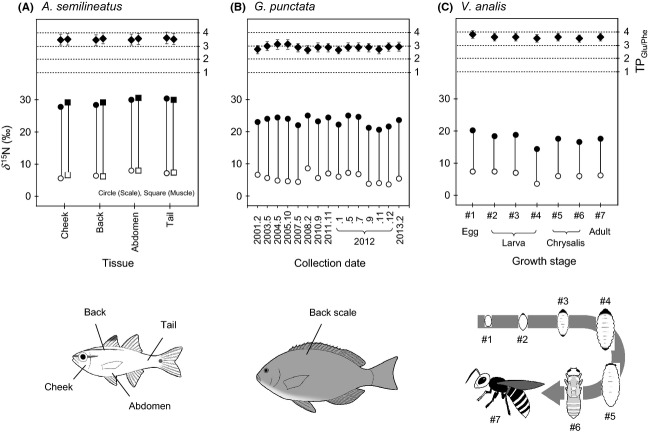
*δ*^15^N values of glutamic acid and phenylalanine and the TP_G__lu/Phe_ values for (A) difference parts (cheek, back, abdomen, and tail) and tissues (scale and muscle) within a single fish *Apogon semilineatus*, (B) different individuals of a fish *Girella punctata* collected during 2001–2013, and (C) different growth stages of a hornet *Vespa analis*. Bar represents potential uncertainly in TP_Glu/Phe_ calculated by taking into account the propagation of 1*σ* for *δ*^15^N_Glu_, *δ*^15^N_Phe_, *β*, and TDF in eq. (1).

Based on the analysis of 5–15 individuals within a single stage for 11 representative species (i.e., eight coastal marine and three terrestrial organisms, Table 2), we first evaluated natural variation in the TP_Glu/Phe_ value for the investigated organisms. As summarized in Table 2, the standard deviation for the comparison of the TP_Glu/Phe_ values and an average of potential uncertainty in the TP_Glu/Phe_ value calculated by taking into account the propagation of uncertainty on each factor in eq. (1) were always less than 0.13 and 0.46 for coastal marine and less than 0.11 and 0.24 for terrestrial organisms. These were almost identical to the precision levels previously reported for the TP_Glu/Phe_ value (Chikaraishi et al. 2009, 2011). As shown in Fig. 3A, there was a quite small difference in the TP_Glu/Phe_ value (1*σ* = 0.06 for the comparison of the TP_Glu/Phe_ values) among scale and muscle collected from cheek, back, abdomen, and tail within a single sample of the fish *Apogon semilineatus*, although the *δ*^15^N values of phenylalanine are different, ranging up to 2.4‰ among body parts and 1.1‰ between tissue types. A small difference (1*σ* = 0.13) was also found between 17 individuals of the fish *Girella punctata* collected from this coastal area over a decade during 2001–2013, although its phenylalanine has a variation in the *δ*^15^N value ranging up to 5.0‰ during this term (Fig. 3B).

**Table 2:** The estimated TP_Glu/Phe_ values of 5–17 individuals within a single stage for 11 representative species

			TP_Glu/Phe_		
			
Sample		Number of samples	Average	1σ^1^	1σ^2^
Red algae	*Gelidium japonicum*	5	1.07	0.11	0.15
Gastropod	*Omphalius pfeifferi*	5	2.01	0.09	0.22
Crustacea	*Polistes japonicus*	5	3.86	0.09	0.46
Fish	*Apogon semilineatus*	11	3.53	0.05	0.42
Fish	*Girella punctata*	15	2.88	0.13	0.33
Fish	*Parapristipoma trilineatum*	5	2.91	0.06	0.33
Fish	*Pseudolabrus siebold*	5	3.32	0.07	0.39
Fish	*Sebastiscus marmoratus*	5	4.06	0.13	0.50
Paper wasp	*Polistes rothneyi*	6	3.02	0.09	0.24
Ladybug	*Harmonia axyridis*	5	3.06	0.07	0.24
Ladybug	*Illeis koebelei*	5	3.05	0.11	0.24

1 Standard deviation (1σ) for the comparison of the TP_Glu/Phe_ values from multiple samples.

2 An average of potential uncertainly in TP_Glu/Phe_ value calculated by taking into account the propagation of 1σ for *δ*^15^N_Glu_, *δ*^15^N_Phe_, *β*, and TDF in eq. (1).

Secondly, we evaluated the effect of metamorphosis on the TP_Glu/Phe_ value from the egg to adult stages of terrestrial insect species. We investigated this because the feeding pattern and appearance of many holometabolous insects show a marked change during metamorphosis. As summarized in Table 3, the standard deviation (1*σ*) for the comparison of the TP_Glu/Phe_ values was always less than 0.14 units for seven terrestrial insect species including herbivore (butterfly) and carnivores (paper wasps, ladybug, and hornet). Interestingly, a small change in the TP_Glu/Phe_ value (1*σ* = 0.11) between different stages is commonly found even in the hornet *Vespa analis*, an opportunistic predator (they can feed on many insects; Takamizawa 2005). The constancy in the TP_Glu/Phe_ value of this hornet was evident despite the fact that there were marked differences (between 3.6 and 7.4‰) in the *δ*^15^N values of phenylalanine at different growth stages, which represent temporal changes in the diet of this hornet family (Fig. 3C). These results reveal how a consumer's trophic position can remain unchanged during a given period of time, even though its food type and/or source has changed dramatically.

**Table 3:** Standard deviation (1s) of the estimated TP_Glu/Phe_ values of seven representative terrestrial species with different growth stages

		N						TP_Glu/Phe_	
			
Sample		Egg	Larva	Chrysalis	Adult	Total	Average	1σ^1^	1σ^2^
Butterfly	*Pieris rapae*	0	4	0	2	6	2.09	0.14	0.24
Paper wasp	*Polistes japonicus*	1	2	2	1	6	3.02	0.14	0.24
Paper wasp	*Polistes jokahamae*	1	1	0	1	3	3.07	0.14	0.24
Paper wasp	*Polistes rothneyi*	1	3	5	4	13	3.03	0.14	0.24
Paper wasp	*Parapolybia indica*	0	3	4	2	9	2.97	0.11	0.24
Ladybug	*Harmonia axyridis*	0	1	1	5	6	3.07	0.06	0.24
Hornet	*Vespa analis*	1	3	2	1	7	3.05	0.11	0.29

1 Standard deviation (1σ) for the comparison of the TP_Glu/Phe_ values from multiple samples.

2 An average of potential uncertainly in TP_Glu/Phe_ value calculated by taking into account the propagation of 1σ for *δ*^15^N_Glu_, *δ*^15^N_Phe_, *β*, and TDF in eq. (1).

### Mapping of food webs using trophic isoclines

**Figure 4:**
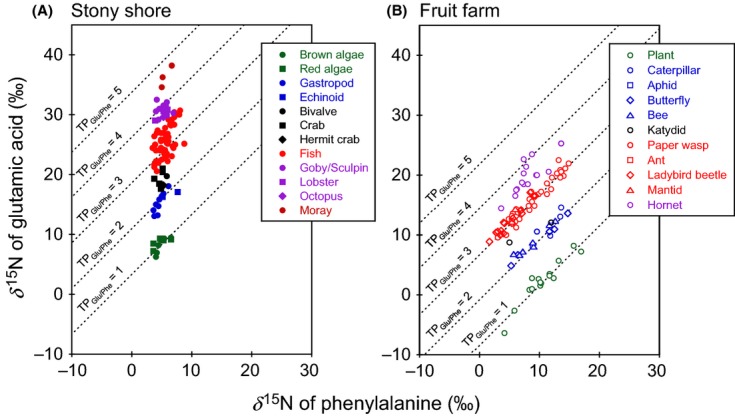
Cross-plots for *δ*^15^N values of glutamic acid and phenylalanine for (A) coastal marine and (B) terrestrial ecosystems. The potential propagation uncertainly is 0.15 for brown and red macroalgae, 0.19–0.22 for gastropod and echinoid, 0.25–0.29 for bivalve, crab, and hermit crab, 0.30–0.42 for fish, 0.43–0.53 for goby/sculpin, lobster, and octopus, 0.55–0.59 for moray, 0.30–0.36 for plant, 0.23–0.25 for caterpillar, aphid, butterfly, and bee, 0.23–0.24 for katydid, 0.23–0.26 for paper wasp, ant, ladybird beetle, and mantid, and 0.27–0.33 for hornet.

Using equation (1), the *δ*^15^N values for phenylalanine and glutamic acid can be plotted against each other, creating a line for each trophic position with slope of 1.0, and between-line interval of 7.6‰ (Fig. 4). All points within each line are the algebraic solutions for the parameter of the isotopic composition of glutamic acid, while holding the trophic position constant and substituting into the equation a range of phenylalanine *δ*^15^N values. Each line therefore represents a trophic isocline (or a “trophocline”), and altogether, these lines demarcate the trophic levels of a food web in 2-dimensional phase space. In this space, the trophic position of organisms can be plotted according to their respective *δ*^15^N values of glutamic acid and phenylalanine. One of the advantages of this graphical presentation is that background heterogeneity in the isotopic composition is completely transparent (evident as the *δ*^15^N value of phenylalanine along the horizontal axis). Whatever the *δ*^15^N values of phenylalanine in an organism are, the *δ*^15^N value of glutamic acid will reflect its trophic position. When the TP_Glu/Phe_ values of organisms are arrayed across trophoclines in phase space, it becomes apparent how populations simultaneously vary in terms of trophic position and background *δ*^15^N values (e.g., Chikaraishi et al. 2009). For example, the isotopic composition of phenylalanine is highly variable in the coastal marine and terrestrial ecosystems (the *δ*^15^N values ranging from 3.5 to 8.7‰ and from 1.6 to 17.0‰, respectively). Despite this high level of background heterogeneity, all of the algal and higher plant samples have the TP_Glu/Phe_ values that were on or near the line of TP_Glu/Phe_ = 1 (Fig. 4), within the precision levels (e.g., 0.15 unit for aquatic algae and 0.30–0.36 unit for terrestrial plants, as potential uncertainty in the TP_Glu/Phe_ value) in coastal marine (*χ*^2^ = 49.994, df = 11, *P* = 1.000) and terrestrial environments (*χ*^2^ = 64.330, df = 14, *P* = 1.000). Furthermore, the species known to be herbivores, such as the gastropods, caterpillars, and bees, all were plotted on the TP_Glu/Phe_ = 2 line within the precision levels (e.g., 0.19–0.22 unit for aquatic and 0.23–0.25 unit for terrestrial organisms, as potential uncertainty in the TP_Glu/Phe_ value) in coastal marine (*χ*^2^ = 70.314, df = 10, *P* = 1.000) and terrestrial environments (*χ*^2^ = 54.757, df = 18, *P* = 1.000).

Importantly, the array of data points in this phase space could reveal linear food chains within the broader food web. Considering that the TDF value for phenylalanine is only 0.4 ± 0.5‰ (Chikaraishi et al. 2009), the *δ*^15^N values of phenylalanine in a consumer closely reflect those of all the resources (e.g., Chikaraishi et al. 2009). In other words, consumer and resource species arrayed in vertical columns within a narrow range of the *δ*^15^N values of phenylalanine could represent highly compartmentalized and linear food webs, whereas a species that registers a wide range of the *δ*^15^N value of phenylalanine could indicate a consumer that can exploit resources from multiple communities, ecosystems, or bioregions. Also, all consumer species falling within a range of *δ*^15^N values for phenylalanine may effectively “belong” to a single particular food web. In fact, in the present study, the *δ*^15^N values of phenylalanine of the algae in the coastal marine system ranged from 3.6 to 6.6‰, which corresponds very closely to the range found in coastal marine consumers (from 3.5 to 8.7‰) (Fig. 4). In the terrestrial system, the *δ*^15^N values of phenylalanine in plants ranged from 4.1 to 17.0‰, which was more variable but nevertheless corresponded closely to the range found in terrestrial consumers (1.6 to 14.9‰) (Fig. 4). These results suggest that the consumer species of each ecosystem had likely fed principally on the local resources and thus were derived from these particular food webs.

**Figure 5:**
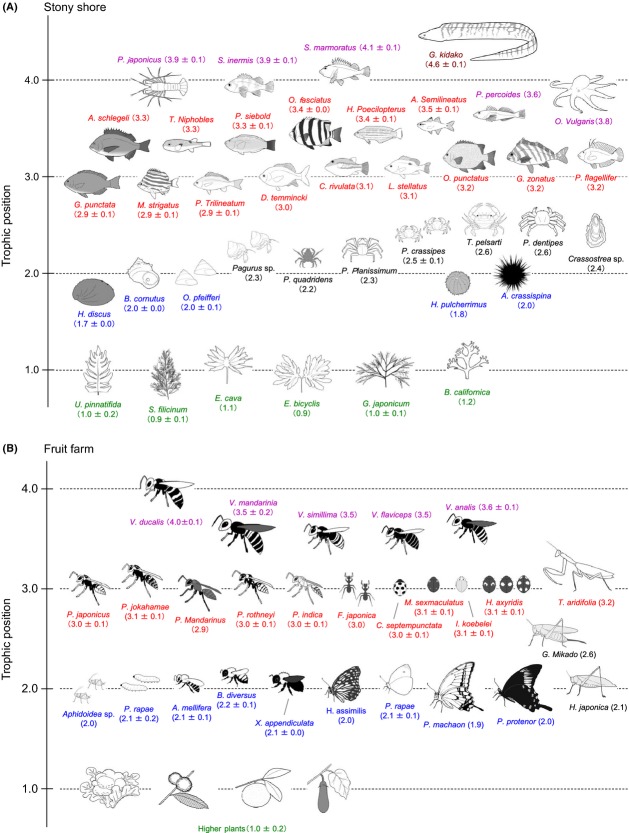
Illustration of food web structure in (A) the coastal marine and (B) terrestrial ecosystems. Mean trophic position and 1*σ* for the comparison of the observed TP_Glu/Phe_ values in each species are shown in a parenthesis under each organism.

Most food chains start with primary producers (TP = 1) such as algae and plants, which are eaten by herbivores (strict plant-feeders: TP = 2) and omnivores (both plant- and animal-feeders: TP > 2). Herbivores and omnivores are eaten by carnivores (animal-feeders: TP > 3) and finally by tertiary predators (carnivores at the top of the food chain). Based on the observed TP_Glu/Phe_ values, we can effectively map subsets of the communities within coastal marine (Fig. 5A) and terrestrial ecosystems (Fig. 5B). Marine primary producers were represented by macroalgae with TP_Glu/Phe_ values ranging from 0.9 to 1.2. As expected, gastropods and echinoids registered as herbivores, given TP_Glu/Phe_ values of 1.7 to 2.0. Various crabs and bivalves (i.e., oysters) appear to be omnivores, as their TP_Glu/Phe_ values range from 2.2 to 2.6. On the other hand, fish and lobsters have a large variation in the TP_Glu/Phe_ values, ranging from 2.9 to 4.6, revealing a high degree of trophic omnivory within this group. The moray eel (*Gymnothorax kidako*) appears to be a top predator with a TP_Glu/Phe_ value of 4.6 in this environment.

In the farm ecosystem (Fig. 5B), higher plants had TP_Glu/Phe_ values ranging from 0.7 to 1.3. The data are consistent with the ecologically expected trophic positions for aphids (*Aphidoidea* sp., TP_Glu/Phe_ = 2.0), caterpillars (*Pieris rapae*, TP_Glu/Phe_ = 2.1), bees (e.g., *Apis mellifera*, TP_Glu/Phe_ = 2.1), butterflies (e.g., *P. rapae*, TP_Glu/Phe_ = 2.1), and herbivorous katydids (*Holochlora japonica*, TP_Glu/Phe_ = 2.1), all of which are known herbivores. *Gampsocleis mikado*, a katydid species known to be an omnivorous scavenger (e.g., ElEla et al. 2010), registered a TP_Glu/Phe_ value of 2.6. Paper wasps (e.g., *Polistes japonicusm* TP_Glu/Phe_ = 3.0), ants (*Formica japonica,* TP_Glu/Phe_ = 3.0), ladybird beetles (e.g., *Coccinella septempunctata*, TP_Glu/Phe_ = 3.0), and mantids (*Tenodera aridifolia,* TP_Glu/Phe_ = 3.2) are secondary consumers with TP_Glu/Phe_ values ranging from 2.9 to 3.2. The TP_Glu/Phe_ values of hornets (e.g., *V. analis* and *Vespa ducalis*) ranged from 3.5 to 4.0.

Trophic omnivory among carnivorous species can be measured as the degree to which consumers’ trophic positions depart from an integer-based trophic position (i.e., trophic level 3.0, 4.0). For example, the mean TP_Glu/Phe_ value of carnivorous/omnivorous fish was 3.33 ± 0.47, which was significantly different from trophic level 3.0 (one-sample *t*-test: *t* = 5.59, df = 62, *P* < 0.001) or 4.0 (*t* = −11.34, df = 62, *P* < 0.001). The value of hornets was 3.64 ± 0.06), which was significantly different from either trophic level 3.0 (*t* = 11.45, df = 14, *P* < 0.001) or 4.0 (*t* = −6.44, df = 14, *P* < 0.001).

In the present study, the trophic position was calculated using eq. (1) with the *β* value of −3.4‰ for coastal marine and +8.4‰ for terrestrial samples and with the TDF value of 7.6‰ for both ecosystems, according to Chikaraishi et al. (2010). On the other hand, recent studies also reported potential variation in the *β* and TDF values for several species, which may leads under- or over-estimation of the trophic position of organisms by up to 2.0 unit (e.g., Germain et al. 2013; Vander Zanden et al. 2013). However, it seems to be that the *β* and TDF values reported in Chikaraishi et al. (2010) are applicable in the studied food webs. In fact, the estimated TP_Glu/Phe_ values of primary producers (i.e., macroalgae and plants) and herbivores (e.g., gastropods and caterpillars) were always close to 1.0 and 2.0, respectively, within the precision levels (Fig. 5). The TP_Glu/Phe_ values of wasps (2.9–3.0) and a hornet *V. ducalis* (4.0) are particularly consistent with the biologically expected trophic positions that the wasps feed primarily on caterpillars found on plant leaves and this hornet feeds solely on wasps (e.g., Takamizawa 2005).

## Implications

In the traditional approach to the trophic position estimation using bulk *δ*^15^N values of organisms, substantial background heterogeneity in the isotopic composition often causes significant uncertainty in the mapping of food web structure (e.g., Cabana and Rasmussen 1996; Vander Zanden et al. 1997; Post 2002). The present study demonstrates that *δ*^15^N analysis of individual amino acids can attend to background heterogeneity while simultaneously allowing precise estimation of the trophic positions of free-roaming organisms. As predicted by theory and early empirical work (Polis 1991; Polis and Strong 1996), the trophic structure evident in the marine and terrestrial systems we studied are indicative of multichannel omnivory: A number of the animal species registered noninteger trophic levels. Our data therefore represent evidence of the ubiquity of trophic omnivory in marine and terrestrial ecosystems. Plotting the trophic spectra of these species across trophoclines reveals the degree of omnivory (Fig. 5). Accommodating background heterogeneity and trophic position simultaneously will allow researchers to assess compartmentalization within a food web while also assessing the trophic niche breadth of populations and communities.

Dual isotope analysis using nitrogen (*δ*^15^N) and carbon (*δ*^13^C) in bulk samples has widely been used for the food web structure analysis in a number of previous studies (e.g., Cabana and Rasmussen 1996; Yoshii et al. 1999; Aita et al. 2011). In these studies, ideally, the *δ*^15^N values provide trophic position estimates of organisms because of the significant enrichment in ^15^N with each trophic level (by ∼3‰ at each level; DeNiro and Epstein 1981; Minagawa and Wada 1984), whereas the *δ*^13^C values directly provide diet resources of organisms because of relatively small enrichment along the trophic level (by ∼1‰ at each level; DeNiro and Epstein 1978). Although the carbon isotope analysis of amino acids is still under development (e.g., Corr et al. 2007; Smith et al. 2009; Dunn et al. 2011), little or no trophic enrichment in ^13^C was commonly found in the essential amino acids in controlling feeding experiments (e.g., Hare et al. 1991; O'Brien et al. 2002; Howland et al. 2003; McMahon et al. 2010). Moreover, the *δ*^13^C values in the essential AAs potentially provide taxonomic (e.g., among bacteria, fungi, microalgae, seagrasses, and terrestrial plants; Larsen et al. 2009, 2013) and geographical discrimination among food sources (McMahon et al. 2012). Accordingly, it is expected that the combination of accurate trophic position estimates (using *δ*^15^N values of amino acids) with accurate food source estimates (using *δ*^13^C values of amino acids) will be potentially useful for better understanding the complex networks of multiple food chains.

## Appendix A1: Nitrogen isotopic composition of amino acids in coastal marine organisms

**Table:**

				*δ*^15^N^1^													
							
Sample	Tissue	Collection Date	*δ*^13^C_Bulk_	Bulk	Alanine	Glycine	Valine	Leucine	Isoleucine	Proline	Serine	Methionine	Glutamic acid	Phenylalanine	TP_Glu/Phe_^2^	1*σ*^3^	Source^4^
Macroalgae (Brown algae)																	
*Undaria pinnatifida* (#1)	Whole	2001.2	−10.8	5.6	8.0	−0.7	6.8	5.1	5.7	5.4	−2.1	2.0	6.9	4.1	0.9	0.2	Ref 1
*Undaria pinnatifida* (#2)	Whole	2006.5	−13.8	5.6	7.7	3.2	8.3	8.1	7.2	6.7	−0.3	2.7	9.2	4.7	1.1	0.2	Ref 2
*Sargassum filicinum* (#1)	Whole	2001.2	−15.0	5.6	7.3	−1.5	8.4	2.8	5.8	7.2	−1.6	2.0	6.3	4.1	0.8	0.2	Ref 1
*Sargassum filicinum* (#2)	Whole	2006.4	−14.0	5.9	7.9	−0.8	9.3	4.6	7.2	9.0	1.6	2.5	8.2	4.4	1.0	0.2	Ref 1
*Ecklonia cava*	Whole	2010.9	−17.7	4.8	8.2	2.7	9.4	7.9	7.8	9.2	2.0	4.6	9.3	5.4	1.1	0.2	This study
*Eisenia bicyclis*	Whole	2010.9	n.d.	n.d.	9.3	3.1	10.0	9.1	8.2	8.8	−1.6	3.8	9.4	6.5	0.9	0.2	This study
Macroalgae (Red algae)																	
*Binghamia californica*	Whole	2001.2	−11.8	6.5	6.9	−0.8	8.3	4.9	6.4	7.3	−1.4	0.9	8.6	3.6	1.2	0.2	Ref 1
*Gelidium japonicum* (#1)	Whole	2001.2	−16.9	6.5	8.2	2.9	7.7	5.7	7.0	7.3	−3.3	3.2	9.3	4.7	1.2	0.2	Ref 1
*Gelidium japonicum* (#2)	Whole	2005.5	n.d.	n.d.	8.3	0.6	6.5	5.7	6.2	6.4	−2.5	3.5	9.1	5.2	1.1	0.2	This study
*Gelidium japonicum* (#3)	Whole	2006.5	−12.8	7.3	10.4	0.4	10.6	8.5	9.4	10.5	0.6	3.8	9.2	6.6	0.9	0.2	Ref 1
*Gelidium japonicum* (#4)	Whole	2010.9	−8.7	5.4	8.9	1.5	6.9	7.0	5.9	5.0	n.d.	n.d.	7.3	3.7	1.0	0.2	This study
*Gelidium japonicum* (#5)	Whole	2012.11	n.d.	n.d.	7.4	−0.5	5.1	4.4	8.0	8.8	n.d.	n.d.	9.1	5.1	1.1	0.2	This study
Gastropod																	
*Batillus cornutus* (#1)	Muscle	2001.2	−16.4	7.8	16.6	4.8	15.1	12.7	14.3	14.1	7.0	3.1	16.4	5.0	2.0	0.2	Ref 1
*Batillus cornutus* (#2)	Shell	2001.2	n.d.	n.d.	16.2	3.9	14.9	12.9	14.7	n.d.	7.7	n.d.	15.9	4.6	2.0	0.2	This study
*Haliotis discus* (#1)	Muscle	2001.2	−13.2	6.0	12.6	2.8	12.7	6.1	9.5	12.9	−0.6	1.9	13.2	4.3	1.7	0.2	Ref 1
*Haliotis discus* (#2)	Shell	2001.2	n.d.	n.d.	12.7	2.6	12.4	5.5	9.3	12.7	0.1	n.d.	13.1	3.8	1.8	0.2	This study
*Omphalius pfeifferi* (#1)	Muscle	2001.2	−14.4	7.4	15.1	3.6	11.4	8.9	9.0	11.7	2.4	2.5	14.7	4.4	1.9	0.2	Ref 1
*Omphalius pfeifferi* (#2)	Shell	2001.2	n.d.	n.d.	15.4	3.0	11.7	8.5	9.8	11.1	3.3	n.d.	15.0	4.0	2.0	0.2	This study
*Omphalius pfeifferi* (#3)	Muscle	2006.4	n.d.	n.d.	15.2	5.1	13.5	12.3	16.7	13.0	4.1	4.3	18.0	6.1	2.1	0.2	This study
*Omphalius pfeifferi* (#4)	Muscle	2010.11	n.d.	n.d.	13.6	2.5	13.8	11.2	12.6	13.3	4.1	1.7	14.0	3.6	1.9	0.2	This study
*Omphalius pfeifferi* (#5)	Muscle	2010.11	n.d.	n.d.	17.0	5.0	16.1	14.2	15.1	15.9	7.4	2.9	16.8	5.2	2.1	0.2	This study
Echinoid																	
*Anthocidaris crassispina*	Shell	2010.11	n.d.	5.8	15.5	6.8	16.0	13.0	12.8	n.d.	n.d.	5.5	16.2	5.2	2.0	0.2	This study
*Hemicentrotus pulcherrimus*	Shell	2010.11	n.d.	7.3	17.6	8.3	15.5	14.3	13.8	n.d.	n.d.	n.d.	17.1	7.6	1.8	0.2	This study
Bivalve																	
*Crassostrea* sp.	Muscle	2010.11	n.d.	8.3	18.4	10.1	21.2	16.7	18.3	17.4	11.4	n.d.	19.7	5.8	2.4	0.3	This study
Crustacean																	
*Pachygrapsus crassipes* (#1)	Muscle	2001.2	−12.5	7.8	15.9	3.7	15.4	10.7	9.4	15.4	3.3	1.9	19.3	3.8	2.6	0.3	Ref 1
*Pachygrapsus crassipes* (#2)	Muscle	2012.7	n.d.	n.d.	17.0	3.1	14.7	14.3	13.7	n.d.	n.d.	n.d.	18.5	4.6	2.4	0.3	This study
*Pagurus filholi*	Muscle	2012.7	n.d.	n.d.	16.0	3.2	13.2	13.0	10.5	n.d.	7.5	3.0	18.2	5.3	2.3	0.2	This study
*Panulirus japonicus* (#1)	Sell	2011.1	−16.7	9.2	25.4	12.1	23.3	21.4	21.0	13.8	5.2	n.d.	29.1	3.9	3.9	0.5	This study
*Panulirus japonicus* (#2)	Sell	2011.1	n.d.	n.d.	25.5	6.2	23.0	19.8	18.8	15.4	7.1	n.d.	30.9	6.0	3.8	0.5	This study
*Panulirus japonicus* (#3)	Sell	2011.1	n.d.	n.d.	25.8	11.2	22.1	21.8	22.3	13.3	5.8	n.d.	29.9	5.8	3.7	0.4	This study
*Panulirus japonicus* (#4)	Sell	2011.1	n.d.	n.d.	23.0	7.6	22.6	23.9	21.4	11.3	4.7	n.d.	31.0	5.1	4.0	0.5	This study
*Panulirus japonicus* (#4)	Sell	2012.5	n.d.	n.d.	27.2	8.8	26.1	24.3	22.2	11.5	3.3	n.d.	30.6	5.2	3.9	0.5	This study
*Plagusia dentipes*	Muscle	2001.2	−13.8	10.1	14.9	6.0	16.3	13.0	9.9	16.9	6.4	2.6	20.4	5.1	2.6	0.3	Ref 1
*Percnon planissimum*	Muscle	2001.2	−12.5	8.4	14.1	7.5	12.8	12.9	12.3	16.4	6.6	1.8	17.9	4.9	2.3	0.2	Ref 1
*Pugettia quadridens*	Muscle	2013.4	n.d.	n.d.	17.4	4.7	15.4	7.9	8.0	16.4	2.2	n.d.	21.0	5.1	2.6	0.3	This study
*Thalamita pelsarti Montgomery*	Muscle	2013.4	n.d.	n.d.	16.4	2.1	13.3	11.3	13.9	13.8	0.7	n.d.	17.7	4.8	2.2	0.2	This study
Fish																	
*Acanthopagrus schlegeli*	Scale	2007.5	−12.2	11.1	20.0	7.4	19.9	19.4	21.5	21.9	11.2	2.4	25.6	4.9	3.3	0.4	Ref 1
*Apogon semilineatus* (*#*1)	Scale	2012.5	−12.7	11.9	25.5	7.5	22.6	23.6	21.7	21.2	n.d.	n.d.	26.4	3.8	3.5	0.4	This study
*Apogon semilineatus* (*#*2)	Scale	2012.5	n.d.	n.d.	25.9	6.7	24.2	22.9	19.5	23.1	n.d.	n.d.	27.0	4.2	3.6	0.4	This study
*Apogon semilineatus* (*#*3)	Scale	2012.5	n.d.	n.d.	27.2	8.8	25.5	24.2	23.2	22.5	n.d.	n.d.	27.4	4.2	3.6	0.4	This study
*Apogon semilineatus* (*#*4–1)	Scale	2012.5	n.d.	n.d.	27.0	7.2	24.6	23.4	20.5	24.5	10.6	4.4	27.7	5.5	3.5	0.4	This study
*Apogon semilineatus* (*#*4–2)	Scale	2012.5	n.d.	n.d.	26.4	6.9	26.2	23.4	23.5	22.5	n.d.	n.d.	28.4	6.4	3.5	0.4	This study
*Apogon semilineatus* (*#*4–3)	Scale	2012.5	n.d.	n.d.	24.7	6.1	24.3	22.4	23.6	24.7	n.d.	n.d.	30.1	7.9	3.5	0.4	This study
*Apogon semilineatus* (*#*4–4)	Scale	2012.5	n.d.	n.d.	25.3	6.6	23.6	24.4	20.6	26.7	9.1	6.4	30.4	7.1	3.6	0.4	This study
*Apogon semilineatus* (*#*4–5)	Muscle	2012.5	n.d.	n.d.	25.5	8.5	24.8	22.8	24.3	24.0	4.1	4.5	29.1	6.6	3.5	0.4	This study
*Apogon semilineatus* (*#*4–6)	Muscle	2012.5	n.d.	n.d.	25.5	8.0	24.7	19.9	21.5	24.9	3.5	4.9	29.1	6.1	3.6	0.4	This study
*Apogon semilineatus* (*#*4–7)	Muscle	2012.5	n.d.	n.d.	24.3	7.6	22.4	18.3	23.9	23.1	6.5	4.6	30.7	8.0	3.5	0.4	This study
*Apogon semilineatus* (*#*4–8)	Muscle	2012.5	n.d.	n.d.	24.6	6.1	22.2	19.1	25.7	25.9	5.7	3.3	30.0	7.3	3.5	0.4	This study
*Canthigaster rivulata*	Muscle	2012.11	−13.8	12.3	21.2	2.1	19.8	21.0	20.9	n.d.	n.d.	n.d.	25.5	6.1	3.1	0.4	This study
*Ditrema temmincki temmincki*	Scale	2012.11	−12.9	11.0	21.6	6.0	22.3	17.7	18.1	22.5	n.d.	n.d.	23.8	5.5	3.0	0.3	This study
*Girella punctata* (#1)	Scale	2001.2	n.d.	n.d.	19.3	6.5	22.4	16.4	19.7	19.3	n.d.	n.d.	23.1	6.6	2.7	0.3	This study
*Girella punctata* (#2)	Scale	2003.5	n.d.	n.d.	19.6	6.2	21.8	19.3	18.7	22.5	n.d.	n.d.	24.0	5.6	3.0	0.3	This study
*Girella punctata* (#3)	Scale	2004.5	n.d.	n.d.	19.4	6.3	22.4	16.5	20.0	23.2	n.d.	n.d.	24.4	4.9	3.1	0.4	This study
*Girella punctata* (#4)	Scale	2005.10	n.d.	n.d.	19.2	6.3	21.8	19.0	19.3	21.7	n.d.	n.d.	24.0	4.7	3.1	0.4	This study
*Girella punctata* (#5)	Scale	2007.5	−13.8	11.1	20.7	6.3	20.2	18.1	19.8	19.4	11.5	1.6	22.0	4.4	2.9	0.3	Ref 1
*Girella punctata* (#6)	Scale	2008.2	n.d.	n.d.	19.5	6.5	21.1	18.6	22.0	19.2	n.d.	n.d.	25.1	8.7	2.7	0.3	This study
*Girella punctata* (#7)	Scale	2010.9	−13.9	11.8	23.5	8.2	26.2	24.6	24.5	25.4	3.9	4.4	23.2	5.6	2.9	0.3	This study
*Girella punctata* (#8)	Scale	2011.11	−14.7	11.1	24.2	7.9	22.4	19.9	19.3	23.2	9.6	6.4	24.5	7.0	2.9	0.3	This study
*Girella punctata* (#9)	Scale	2012.1	n.d.	n.d.	20.2	7.0	21.0	19.7	18.6	18.5	n.d.	n.d.	22.2	6.0	2.7	0.3	This study
*Girella punctata* (#10)	Scale	2012.5	n.d.	n.d.	19.5	7.6	20.7	19.1	20.3	19.2	n.d.	n.d.	25.0	7.2	2.9	0.3	This study
*Girella punctata* (#11)	Scale	2012.7	n.d.	n.d.	17.9	10.5	20.0	n.d.	19.3	n.d.	n.d.	n.d.	24.7	6.8	2.9	0.3	This study
*Girella punctata* (#12)	Scale	2012.9	n.d.	n.d.	21.2	7.5	22.2	27.3	16.5	15.4	9.7	1.6	21.3	3.7	2.9	0.3	This study
*Girella punctata* (#13)	Scale	2012.11	n.d.	n.d.	20.0	9.6	18.7	15.5	12.1	18.4	n.d.	2.2	20.5	4.1	2.7	0.3	This study
*Girella punctata* (#14)	Scale	2012.12	n.d.	n.d.	20.4	8.7	17.0	16.9	19.8	20.0	n.d.	1.5	21.7	3.7	2.9	0.3	This study
*Girella punctata* (#15)	Scale	2013.2	n.d.	n.d.	16.6	4.9	19.2	18.9	20.0	n.d.	n.d.	n.d.	23.5	5.4	2.9	0.3	This study
*Gymnothorax kidako* (#1)	Muscle	2011.11	−14.2	13.9	36.2	10.3	34.1	27.1	31.3	31.5	5.8	2.6	36.2	5.2	4.6	0.6	This study
*Gymnothorax kidako* (#2)	Muscle	2012.2	−14.6	13.4	32.5	9.8	33.1	32.3	38.3	15.5	n.d.	4.1	38.2	6.6	4.7	0.6	This study
*Gymnothorax kidako* (#3)	Muscle	2012.11	n.d.	n.d.	28.5	7.6	28.2	25.4	37.3	29.1	n.d.	2.7	34.6	5.0	4.4	0.6	This study
*Goniistius zonatus*	Scale	2012.11	−12.3	10.3	21.0	7.9	22.2	17.6	19.2	23.1	n.d.	5.4	26.2	6.1	3.2	0.4	This study
*Halichoeres poecilopterus* (#1)	Scale	2010.9	−11.6	12.3	21.4	11.0	20.6	20.0	11.6	25.6	n.d.	n.d.	27.0	5.3	3.4	0.4	This study
*Halichoeres poecilopterus* (#2)	Scale	2011.12	n.d.	n.d.	23.7	7.5	24.7	22.9	22.9	27.6	n.d.	5.5	28.3	7.2	3.3	0.4	This study
*Halichoeres poecilopterus* (#3)	Scale	2011.12	n.d.	n.d.	23.7	9.5	24.5	22.6	18.0	25.7	n.d.	6.4	29.6	7.1	3.5	0.4	This study
*Lutjanus stellatus*	Scale	2012.11	−10.1	10.6	21.9	9.0	21.3	16.3	16.5	21.5	n.d.	4.7	25.2	6.0	3.1	0.4	This study
*Microcanthus strigatus* (#1)	Scale	2011.11	−13.4	12.0	20.6	7.0	22.9	22.2	22.0	20.8	11.8	4.6	24.1	5.3	3.0	0.3	This study
*Microcanthus strigatus* (#2)	Scale	2012.1	n.d.	n.d.	22.4	7.2	22.9	24.0	21.1	22.7	n.d.	n.d.	22.8	5.8	2.8	0.3	This study
*Microcanthus strigatus* (#3)	Scale	2012.7	n.d.	n.d.	21.5	19.5	21.5	20.1	19.7	18.7	n.d.	3.9	22.4	5.0	2.8	0.3	This study
*Oplegnathus fasciatus* (#1)	Scale	2012.8	−13.3	12.8	23.9	3.6	21.5	20.0	19.1	n.d.	8.9	3.3	25.1	3.9	3.3	0.4	This study
*Oplegnathus fasciatus* (#2)	Scale	2012.8	−13.6	12.6	27.0	9.6	23.6	25.2	22.3	n.d.	12.4	5.0	28.2	6.6	3.4	0.4	This study
*Oplegnathus punctatus*	Scale	2012.9	−11.8	11.1	18.6	6.3	18.0	13.6	14.1	12.7	10.6	3.3	24.1	4.0	3.2	0.4	This study
*Parapristipoma trilineatum* (#1)	Scale	2012.9	−12.8	12.4	21.7	8.1	20.0	16.8	12.7	14.4	10.5	3.2	22.3	4.5	2.9	0.3	This study
*Parapristipoma trilineatum* (#2)	Scale	2012.9	n.d.	n.d.	22.1	8.3	17.5	15.7	12.0	11.6	11.5	4.8	23.1	5.7	2.8	0.3	This study
*Parapristipoma trilineatum* (#3)	Scale	2012.9	n.d.	n.d.	21.3	8.9	19.5	13.6	10.2	20.6	9.5	3.9	22.3	4.1	2.9	0.3	This study
*Parapristipoma trilineatum* (#4)	Scale	2012.9	n.d.	n.d.	22.4	8.9	20.6	16.4	19.5	18.7	12.8	4.2	23.4	5.8	2.9	0.3	This study
*Parapristipoma trilineatum* (#5)	Scale	2012.9	n.d.	n.d.	22.8	8.4	20.3	13.9	13.3	17.8	11.8	2.9	24.0	5.4	3.0	0.3	This study
*Pseudoblennius percoides*	Muscle	2011.12	−14.5	14.2	27.7	7.7	28.8	25.0	29.6	29.5	n.d.	5.6	30.2	6.9	3.6	0.4	This study
*Pseudolabrus siebold* (#1)	Scale	2011.11	−11.3	11.5	21.0	8.2	21.4	17.6	18.7	22.4	11.2	2.4	24.4	3.5	3.3	0.4	This study
*Pseudolabrus siebold* (#2)	Scale	2011.12	n.d.	n.d.	23.6	10.2	24.3	22.1	22.3	25.2	15.2	4.9	26.8	5.9	3.3	0.4	This study
*Pseudolabrus siebold* (#3)	Scale	2011.12	n.d.	n.d.	23.1	8.9	25.7	22.6	18.6	n.d.	n.d.	n.d.	25.8	5.3	3.2	0.4	This study
*Pseudolabrus siebold* (#4)	Scale	2011.12	n.d.	n.d.	24.2	8.7	26.3	24.0	21.9	23.8	n.d.	n.d.	25.8	4.9	3.3	0.4	This study
*Pseudolabrus siebold* (#5)	Scale	2013.2	n.d.	n.d.	24.7	7.9	24.7	25.7	21.3	n.d.	n.d.	n.d.	26.9	4.9	3.4	0.4	This study
*Pteragogus flagellifer*	Scale	2011.12	−14.4	11.8	26.4	12.9	21.6	17.8	n.d.	24.5	n.d.	5.6	26.0	5.7	3.2	0.4	This study
*Sebastes inermis* (#1)	Scale	2012.5	−12.5	12.5	28.4	6.4	30.5	24.3	27.4	31.6	8.9	3.2	31.6	5.3	4.0	0.5	This study
*Sebastes inermis* (#2)	Scale	2013.2	n.d.	n.d.	23.2	3.0	25.8	19.3	23.6	30.2	2.8	n.d.	29.2	4.4	3.8	0.5	This study
*Sebastiscus marmoratus* (#1)	Scale	2012.1	−13.1	13.1	29.4	8.9	27.9	28.4	29.6	30.1	13.7	1.8	30.6	4.0	4.0	0.5	This study
*Sebastiscus marmoratus* (#2)	Scale	2012.1	−11.6	12.4	30.1	7.1	28.1	31.1	30.7	31.4	11.9	3.8	32.6	4.2	4.3	0.5	This study
*Sebastiscus marmoratus* (#3)	Scale	2012.1	n.d.	n.d.	31.7	7.8	28.0	32.6	26.9	30.0	13.5	4.9	32.1	5.8	4.0	0.5	This study
*Sebastiscus marmoratus* (#4)	Scale	2012.1	n.d.	n.d.	28.2	7.5	26.2	29.9	25.6	28.5	n.d.	n.d.	30.9	4.5	4.0	0.5	This study
*Sebastiscus marmoratus* (#5)	Scale	2013.2	n.d.	n.d.	28.1	7.8	29.3	22.7	24.9	n.d.	n.d.	n.d.	31.5	5.7	3.9	0.5	This study
*Takifugu niphobles*	Muscle	2010.9	−15.8	12.9	24.9	11.5	n.d.	26.0	21.9	n.d.	17.4	3.5	26.2	5.7	3.3	0.4	This study
Octopus																	
*Octopus vulgaris*	Muscle	2013.1	n.d.	n.d.	22.5	6.0	26.1	21.3	22.7	n.d.	1.0	n.d.	29.6	5.3	3.8	0.5	This study

n.d.: Not determined.

1 The *δ*^15^N value was determined by single analysis for each sample.

2 TP_Glu/Phe_ = (*δ*^15^N_Glu_ − *δ*^15^N_Phe_ − 3.4)/7.6 + 1.

3 Propagation error on the TP_Glu/Phe_ value based on 1*σ* on the *δ*^15^N measurement of amino acids in this study and 1*σ* on the *β* and TDF values reported in Chikaraishi et al. 2010.

4 Ref 1: Chikaraishi et al. (2009); Ref 2: Chikaraishi et al. (2010).

## Appendix 2: Nitrogen isotopic composition of amino acids in terrestrial organisms

**Table:**

					*δ*^15^N^1^													
								
Sample	Stage	Tissue	Collection Date	*δ*^13^C_Bulk_	Bulk	Alanine	Glycine	Valine	Leucine	Isoleucine	Proline	Serine	Methionine	Glutamic acid	Phenylalanine	TP_Glu/Phe_^2^	1*σ*^3^	Source^4^
Higher plant																		
*Brassica oleracea* (#1)	–	Leaf	2008.11	−32.5	4.7	0.2	−6.7	5.1	3.8	3.9	9.5	1.0	0.8	5.7	13.1	1.1	0.3	Ref 2
*Brassica oleracea* (#2)	–	Leaf	2011.11	n.d.	n.d.	1.7	−7.0	3.7	0.7	3.4	4.6	−2.1	n.d.	2.6	9.8	1.2	0.3	Ref 2
*Brassica oleracea* (#3)	–	Leaf	2011.11	n.d.	n.d.	4.3	−8.0	3.4	−1.2	−0.8	6.3	−5.5	n.d.	3.4	11.7	1.0	0.3	Ref 2
*Daucus carota*	–	Leaf	2011.11	−30.6	5.9	8.3	−2.2	8.0	4.6	7.3	7.2	n.d.	n.d.	8.2	15.7	1.1	0.3	Ref 3
*Castanea crenata* (#1)	–	Leaf	2008.11	−30.0	0.7	−2.1	−12.7	−0.3	0.5	1.6	4.6	−4.2	n.d.	1.5	10.1	1.0	0.3	Ref 2
*Castanea crenata* (#2)	–	Leaf	2010.11	−29.3	1.8	0.7	−9.8	0.2	−2.4	−0.7	6.2	n.d.	n.d.	2.9	8.8	1.3	0.3	This study
*Castanea crenata* (#3)	–	Nut	2008.11	n.d.	n.d.	n.d.	−14.8	n.d.	0.1	n.d.	2.9	−7.2	n.d.	0.8	8.3	1.1	0.3	Ref 2
*Citrus unshiu*	–	Leaf	2011.11	−30.6	4.9	6.4	−6.8	4.7	3.1	3.0	8.9	−0.6	n.d.	2.8	12.4	0.8	0.3	Ref 3
*Cucurbita moschata*	–	Leaf	2012.8	−28.3	4.3	3.9	−8.6	1.0	−0.6	0.1	1.0	−14.5	n.d.	2.1	10.1	1.1	0.3	This study
*Diospyros kaki Thunberg*	–	Leaf	2012.6	−29.3	2.8	−1.2	−4.4	−1.2	−2.2	0.0	0.0	−3.3	n.d.	1.0	8.8	1.1	0.3	This study
*Prunus avium*	–	Leaf	2012.6	−30.1	3.8	1.9	−2.9	2.9	2.1	1.2	2.9	−4.3	n.d.	3.2	11.6	1.0	0.3	This study
*Raphanus sativus*	–	Leaf	2011.11	−29.8	4.0	−3.6	−8.4	−2.9	−3.8	−4.3	3.3	−4.9	n.d.	−2.6	5.9	1.0	0.3	Ref 3
*Solanum lycopersicum*	–	Leaf	2011.11	−28.5	5.2	6.2	−3.6	2.0	2.9	1.3	8.6	−4.0	n.d.	2.0	10.3	1.0	0.3	Ref 3
*Solanum melongena*	–	Leaf	2011.11	−27.7	5.6	5.6	−4.6	6.8	1.3	−0.2	15.1	5.1	n.d.	7.2	17.0	0.8	0.4	Ref 3
*Solanum tuberosum*	–	Leaf	2011.11	−29.5	−2.8	−2.4	−12.1	−2.0	−5.7	−3.5	−2.0	n.d.	n.d.	−6.3	4.1	0.7	0.4	Ref 3
Aphid																		
*Aphidoidea* sp.	Adult	Whole	2011.11	−21.8	2.2	5.5	2.9	7.1	3.6	5.1	10.4	1.2	n.d.	8.1	8.9	2.0	0.2	Ref 3
Butterfly																		
*Hestina assimilis*	Adult	Whole	2011.8	n.d.	n.d.	3.9	3.9	13.6	12.6	15.7	17.0	3.1	2.1	10.5	11.7	2.0	0.2	This study
*Papilio machaon* (#1)	Adult	Whole	2011.9	−27.3	2.8	9.1	2.4	11.8	3.6	9.5	14.7	7.5	1.7	11.0	12.5	1.9	0.3	This study
*Papilio protenor*	Adult	Leg	2012.9	−29.1	6.0	12.6	6.6	8.5	7.2	10.2	19.1	6.8	n.d.	13.6	14.7	2.0	0.2	This study
*Pieris rapae* (#1)	Larva	Whole	2008.11	−29.6	1.9	6.6	−0.4	8.2	7.0	8.9	16.3	3.7	1.6	13.0	13.4	2.0	0.2	Ref 2
*Pieris rapae* (#2)	Larva	Whole	2008.11	−26.9	1.9	5.3	−2.7	7.4	6.5	8.5	14.3	2.3	1.0	14.6	13.6	2.2	0.2	Ref 2
*Pieris rapae* (#3)	Larva	Whole	2011.11	n.d.	n.d.	9.1	0.1	9.6	2.7	7.0	13.1	1.6	n.d.	10.6	9.5	2.2	0.2	This study
*Pieris rapae* (#4)	Larva	Whole	2011.11	n.d.	n.d.	8.3	0.8	6.5	2.4	4.6	11.8	1.5	n.d.	9.9	11.9	1.8	0.3	This study
*Pieris rapae* (#5)	Adult	Whole	2011.5	−30.5	1.6	7.7	5.1	6.8	4.0	11.2	12.2	0.9	−7.5	4.8	5.3	2.0	0.2	This study
*Pieris rapae* (#6)	Adult	Whole	2011.5	n.d.	n.d.	5.2	3.1	8.6	6.4	10.5	12.7	1.0	0.1	6.7	6.4	2.1	0.2	This study
Bee																		
*Apis mellifera* (#1)	Adult	Whole	2009.8	−26.8	1.6	4.8	6.5	6.4	0.9	3.2	15.0	2.8	1.4	8.0	9.1	2.0	0.2	Ref 3
*Apis mellifera* (#2)	Adult	Whole	2009.8	n.d.	n.d.	4.1	3.4	3.2	−0.2	0.2	10.1	0.9	n.d.	7.3	7.2	2.1	0.2	Ref 3
*Apis mellifera* (#3)	Adult	Whole	2009.8	n.d.	n.d.	6.3	6.9	7.5	5.8	5.5	20.8	n.d.	3.0	11.7	11.6	2.1	0.2	Ref 3
*Bombus diversus diversus* (#1)	Adult	Whole	2010.10	−26.9	2.2	2.4	2.1	2.0	−0.8	0.3	12.2	n.d.	−0.7	6.9	5.7	2.3	0.2	Ref 3
*Bombus diversus diversus* (#2)	Adult	Whole	2012.5	n.d.	n.d.	5.1	0.0	4.3	3.2	5.2	4.1	−1.1	n.d.	6.7	6.7	2.1	0.2	This study
*Xylocopa appendiculata* (#1)	Adult	Whole	2009.8	−25.0	5.1	9.8	10.3	11.1	11.5	7.8	19.1	6.0	−1.1	12.3	12.6	2.1	0.2	Ref 3
*Xylocopa appendiculata* (#2)	Adult	Whole	2012.4	n.d.	n.d.	6.3	4.2	9.4	5.0	1.0	17.2	4.2	−1.9	8.8	8.8	2.1	0.2	This study
Katydid																		
*Gampsocleis mikado*	Adult	Leg	2012.9	−26.2	2.0	5.5	−0.3	5.0	3.0	9.7	n.d.	n.d.	−2.7	8.8	4.9	2.6	0.2	This study
*Holochlora japonica*	Adult	Whole	2011.11	−26.4	9.1	9.7	4.5	11.2	9.5	12.4	15.6	1.8	1.2	12.1	11.9	2.1	0.2	This study
Paper wasp																		
*Polistes japonicus japonicus* (#1)	Egg	Whole	2010.8	−27.2	4.9	7.9	−1.0	16.4	7.6	10.0	16.7	−1.6	0.0	19.9	14.1	2.9	0.2	Ref 3
*Polistes japonicus japonicus* (#2)	Larva	Whole	2010.8	−29.7	1.6	4.2	2.1	15.7	2.1	5.1	14.0	0.0	−1.5	16.8	9.9	3.0	0.2	Ref 3
*Polistes japonicus japonicus* (#3)	Larva	Whole	2010.8	−30.1	1.1	4.6	1.0	14.4	1.4	5.0	13.6	−0.6	−3.4	17.3	8.7	3.2	0.3	Ref 3
*Polistes japonicus japonicus* (#4)	Chrysalis	Whole	2010.8	−29.4	1.4	3.6	1.5	16.4	5.2	7.4	17.5	−0.5	n.d.	17.3	10.2	3.0	0.2	Ref 3
*Polistes japonicus japonicus* (#5)	Chrysalis	Whole	2010.8	−28.6	2.6	8.8	2.2	15.6	3.7	5.2	15.9	−0.5	n.d.	18.6	11.2	3.1	0.2	Ref 3
*Polistes japonicus japonicus* (#6)	Newly-emerged	Whole	2010.8	−28.8	2.7	9.0	1.2	14.0	8.1	8.9	17.0	−4.1	−0.4	17.7	12.0	2.9	0.2	Ref 3
*Polistes jokahamae jokahamae* (#1)	Egg	Whole	2012.7	−29.3	4.5	10.9	6.5	10.8	5.8	6.1	14.1	n.d.	n.d.	12.7	6.6	2.9	0.2	This study
*Polistes jokahamae jokahamae* (#2)	Larva	Whole	2012.7	−28.7	2.4	14.3	0.9	10.7	8.7	10.3	12.0	n.d.	n.d.	13.7	5.6	3.2	0.2	This study
*Polistes jokahamae jokahamae* (#3)	Adult	Whole	2012.7	−29.9	3.9	13.7	3.0	10.5	10.4	8.9	8.8	n.d.	n.d.	13.1	5.4	3.1	0.2	This study
*Polistes mandarinus*	Adult	Leg	2012.11	−23.7	5.5	7.6	8.0	9.8	8.8	18.9	16.1	7.3	n.d.	12.2	5.9	2.9	0.2	This study
*Polistes rothneyi iwatai* (#1)	Egg	Whole	2008.8	−26.1	7.2	9.0	5.1	16.2	10.6	13.5	18.2	10.1	n.d.	22.5	13.2	3.3	0.3	Ref 3
*Polistes rothneyi iwatai* (#2)	Larva	Whole	2008.8	−27.4	4.6	7.4	2.7	14.3	8.2	12.3	16.3	4.3	n.d.	20.7	13.7	3.0	0.2	Ref 3
*Polistes rothneyi iwatai* (#3)	Larva	Whole	2008.8	−27.2	5.5	8.0	0.9	16.0	9.8	13.6	17.9	1.2	n.d.	20.9	13.5	3.1	0.2	Ref 3
*Polistes rothneyi iwatai* (#4)	Larva	Whole	2008.8	−27.2	5.2	8.3	5.5	15.3	9.0	13.9	18.4	5.8	n.d.	19.8	13.5	2.9	0.2	Ref 3
*Polistes rothneyi iwatai* (#5)	Chrysalis	Whole	2008.8	−29.6	5.5	7.3	2.7	14.3	10.6	12.2	17.3	−0.3	n.d.	20.3	12.9	3.1	0.2	Ref 3
*Polistes rothneyi iwatai* (#6)	Chrysalis	Whole	2008.8	−28.6	5.5	6.0	3.3	15.1	10.0	14.2	18.1	0.8	n.d.	19.5	12.0	3.1	0.2	Ref 3
*Polistes rothneyi iwatai* (#7)	Chrysalis	Whole	2008.8	−29.8	5.4	8.6	3.2	14.0	10.9	13.7	18.4	3.8	n.d.	20.5	13.6	3.0	0.2	Ref 3
*Polistes rothneyi iwatai* (#8)	Chrysalis	Whole	2008.8	−28.1	4.9	9.5	8.5	15.9	12.0	15.3	16.5	1.6	n.d.	21.1	14.4	3.0	0.2	Ref 3
*Polistes rothneyi iwatai* (#9)	Chrysalis	Whole	2008.8	−28.4	4.9	7.8	6.4	15.6	8.4	13.8	18.2	4.7	n.d.	19.6	13.5	2.9	0.2	Ref 3
*Polistes rothneyi iwatai* (#10)	Chrysalis	Whole	2008.8	−28.2	4.9	6.4	10.0	16.7	12.5	15.6	16.7	0.5	n.d.	22.0	14.9	3.0	0.2	Ref 3
*Polistes rothneyi iwatai* (#11)	Newly-emerged	Whole	2008.8	−28.4	2.4	5.5	1.2	11.7	9.7	10.9	14.2	−0.6	n.d.	14.1	8.3	2.9	0.2	Ref 3
*Polistes rothneyi iwatai* (#12)	Adult	Whole	2008.8	−26.8	5.5	12.2	8.4	9.0	5.6	5.6	n.d.	3.0	5.7	11.3	6.2	2.8	0.2	Ref 3
*Polistes rothneyi iwatai* (#13)	Adult	Whole	2009.8	n.d.	n.d.	6.1	5.5	8.8	7.5	11.0	12.6	4.0	−1.8	14.3	6.1	3.2	0.2	Ref 3
*Polistes rothneyi iwatai* (#14)	Adult	Whole	2009.8	n.d.	n.d.	5.9	4.5	8.6	5.6	8.6	13.6	3.0	2.7	15.9	8.4	3.1	0.2	Ref 3
*Parapolybia indica* (#1)	Larva	Whole	2010.8	−27.7	3.5	6.5	4.9	9.5	6.6	8.2	15.9	3.5	n.d.	14.9	9.1	2.9	0.2	Ref 3
*Parapolybia indica* (#2)	Larva	Whole	2010.8	−28.5	2.5	7.6	7.5	11.6	3.5	6.3	15.7	2.8	n.d.	13.8	6.3	3.1	0.2	Ref 3
*Parapolybia indica* (#3)	Larva	Whole	2010.8	−28.6	1.3	7.6	8.6	10.5	4.5	5.5	14.0	2.5	n.d.	12.0	5.1	3.0	0.2	Ref 3
*Parapolybia indica* (#4)	Chrysalis	Whole	2010.8	−28.4	3.7	8.0	8.4	11.1	6.0	9.6	16.2	2.6	n.d.	16.8	11.2	2.8	0.2	Ref 3
*Parapolybia indica* (#5)	Chrysalis	Whole	2010.8	−28.3	4.0	8.2	7.9	12.2	4.7	6.7	15.9	1.3	n.d.	12.7	4.8	3.1	0.2	Ref 3
*Parapolybia indica* (#6)	Chrysalis	Whole	2010.8	−27.6	3.3	7.6	5.8	11.4	5.9	7.2	13.9	3.4	n.d.	9.6	3.0	3.0	0.2	Ref 3
*Parapolybia indica* (#7)	Chrysalis	Whole	2010.8	−28.7	1.4	6.9	5.6	10.0	6.9	7.2	19.5	7.7	n.d.	10.0	4.4	2.8	0.2	Ref 3
*Parapolybia indica* (#8)	Newly-emerged	Whole	2010.8	−27.4	4.9	6.0	2.7	9.8	9.5	9.1	15.5	2.6	n.d.	14.9	8.2	3.0	0.2	Ref 3
*Parapolybia indica* (#9)	Adult	Whole	2010.8	−27.5	4.3	7.8	6.2	10.0	6.9	8.4	15.9	5.4	n.d.	15.6	8.8	3.0	0.2	Ref 3
Ant																		
*Formica japonica*	Adult	Whole	2010.8	n.d.	n.d.	12.7	11.0	18.8	8.3	11.0	12.4	n.d.	n.d.	12.2	5.4	3.0	0.2	This study
Ladybird beetle																		
*Coccinella septempunctata* (#1)	Adult	Whole	2011.5	−27.6	9.1	8.0	5.9	7.5	5.8	8.1	16.8	3.3	0.2	10.1	3.5	3.0	0.2	This study
*Coccinella septempunctata* (#2)	Adult	Whole	2011.10	n.d.	n.d.	8.5	6.6	7.7	6.8	8.4	19.0	n.d.	n.d.	10.8	3.6	3.0	0.2	This study
*Harmonia axyridis* (#1)	Larva	Whole	2011.11	−26.5	6.4	10.8	8.4	9.4	4.9	9.1	18.7	3.7	n.d.	16.5	9.0	3.1	0.2	Ref 3
*Harmonia axyridis* (#2)	Chrysalis	Whole	2011.11	−29.0	4.7	10.2	7.9	10.1	5.0	7.8	15.0	3.7	n.d.	16.6	9.4	3.1	0.2	Ref 3
*Harmonia axyridis* (#3)	Adult	Whole	2011.11	−28.6	7.4	10.9	11.7	9.8	7.4	8.8	23.0	6.6	n.d.	14.2	6.9	3.1	0.2	Ref 3
*Harmonia axyridis* (#4)	Adult	Whole	2012.4	n.d.	n.d.	10.1	6.5	2.3	3.6	5.1	18.9	−9.1	n.d.	10.3	4.0	2.9	0.2	This study
*Harmonia axyridis* (#5)	Adult	Whole	2012.4	n.d.	n.d.	10.9	6.7	5.4	4.4	4.4	19.1	1.4	n.d.	10.6	3.1	3.1	0.2	This study
*Harmonia axyridis* (#6)	Adult	Whole	2012.4	n.d.	n.d.	7.7	5.3		3.9	4.3	6.2	5.9	−8.4	10.5	2.8	3.1	0.2	This study
*Harmonia axyridis* (#6)	Adult	Whole	2012.4	n.d.	n.d.	13.0	10.2	4.2	−0.1	−0.9	19.1	n.d.	n.d.	11.9	4.2	3.1	0.2	This study
*Illeis koebelei* (#1)	Adult	Whole	2012.10	n.d.	n.d.	10.8	6.7	5.6	5.6	8.6	19.1	3.0	n.d.	12.7	5.5	3.1	0.2	This study
*Illeis koebelei* (#2)	Adult	Whole	2012.10	n.d.	n.d.	10.4	6.5	9.3	6.5	8.8	19.4	−1.1	n.d.	12.0	5.5	3.0	0.2	This study
*Illeis koebelei* (#3)	Adult	Whole	2012.11	n.d.	n.d.	11.2	5.3	8.9	7.4	14.0	20.3	n.d.	n.d.	13.6	7.1	3.0	0.2	This study
*Illeis koebelei* (#4)	Adult	Whole	2012.11	n.d.	n.d.	14.8	9.7	13.9	9.2	14.5	n.d.	6.7	1.5	17.0	8.5	3.2	0.3	This study
*Illeis koebelei* (#5)	Adult	Whole	2012.11	n.d.	n.d.	12.6	6.0	12.2	6.3	10.4	n.d.	n.d.	n.d.	14.1	6.8	3.1	0.2	This study
*Menochilus sexmaculatus* (#1)	Adult	Whole	2012.4	n.d.	n.d.	8.4	2.8	5.5	8.0	9.4	n.d.	n.d.	n.d.	8.8	1.6	3.1	0.2	This study
*Menochilus sexmaculatus* (#2)	Adult	Whole	2012.4	n.d.	n.d.	10.3	6.3	10.3	11.4	10.3	n.d.	n.d.	n.d.	12.1	4.1	3.2	0.2	This study
Mantid																		
*Tenodera aridifolia*	Adult	Wing	2012.9	n.d.	n.d.	9.5	7.0	11.0	8.9	11.0	20.7	n.d.	n.d.	14.4	5.9	3.2	0.3	This study
Hornet																		
*Vespa analis fabriciusi* (#1)	Egg	Whole	2011.6	−25.7	6.8	16.9	13.3	20.0	13.7	18.5	23.5	7.7	0.5	20.3	7.4	3.8	0.3	This study
*Vespa analis fabriciusi* (#2)	Larva	Whole	2011.6	−25.6	4.9	13.4	13.0	18.0	11.7	17.2	18.5	7.1	0.6	18.4	7.4	3.6	0.3	This study
*Vespa analis fabriciusi* (#3)	Larva	Whole	2011.6	−25.6	4.5	12.9	14.0	16.9	11.4	16.2	18.5	6.7	0.4	18.8	7.1	3.6	0.3	This study
*Vespa analis fabriciusi* (#4)	Larva	Whole	2011.6	−25.7	3.7	11.4	13.7	15.7	7.8	14.3	16.5	4.9	−1.3	14.5	3.6	3.5	0.3	This study
*Vespa analis fabriciusi* (#5)	Chrysalis	Whole	2011.6	−26.6	4.7	13.4	14.6	18.6	9.9	16.6	18.8	6.0	n.d.	17.6	6.1	3.6	0.3	This study
*Vespa analis fabriciusi* (#6)	Chrysalis	Whole	2011.6	−26.1	5.0	14.3	13.4	17.7	12.1	19.1	20.0	8.3	n.d.	16.6	6.0	3.5	0.3	This study
*Vespa analis fabriciusi* (#7)	Newly-emerged	Whole	2011.6	−26.2	5.0	15.4	15.7	19.9	14.5	17.0	23.5	14.0	−0.3	17.7	6.3	3.6	0.3	This study
*Vespa ducalis pulchra* (#1)	Adult	Whole	2009.8	−25.8	5.4	7.6	5.4	19.9	10.7	13.2	19.1	4.5	n.d.	21.4	7.7	3.9	0.3	Ref 3
*Vespa ducalis pulchra* (#2)	Adult	Whole	2009.8	n.d.	n.d.	10.7	9.5	17.2	10.1	13.2	20.6	6.8	n.d.	23.5	8.8	4.0	0.3	Ref 3
*Vespa ducalis pulchra* (#3)	Adult	Whole	2009.8	n.d.	n.d.	10.0	4.5	14.8	11.0	15.5	16.1	3.0	n.d.	22.6	7.4	4.1	0.3	Ref 3
*Vespa mandarinia japonica* (#1)	Adult	Whole	2010.10	−26.8	4.5	13.2	−0.2	16.8	6.6	8.5	15.3	0.6	1.0	18.5	8.0	3.5	0.3	Ref 3
*Vespa mandarinia japonica* (#2)	Adult	Whole	2012.7	n.d.	n.d.	19.0	1.1	22.2	13.8	10.5	24.3	2.1	−0.1	20.6	11.6	3.3	0.3	This study
*Vespa mandarinia japonica* (#3)	Adult	Whole	2012.7	n.d.	n.d.	23.4	6.5	22.7	14.7	11.5	30.0	3.2	5.3	25.3	13.6	3.6	0.3	This study
*Vespa simillima xanthoptera*	Adult	Whole	2009.8	−25.6	4.9	12.0	7.0	12.2	7.2	10.2	18.3	3.6	n.d.	20.1	9.4	3.5	0.3	Ref 3
*Vespula flaviceps lewisii*	Adult	Whole	2010.10	−24.6	5.6	12.3	5.6	16.6	7.2	10.0	n.d.	5.9	n.d.	20.0	9.7	3.5	0.3	Ref 3

n.d.: Not determined.

1 The *δ*^15^N value was determined by single analysis for each sample.

2 TP_Glu/Phe_ = (*δ*^15^N_Glu_ − *δ*^15^N_Phe_ + 8.4)/7.6 + 1.

3 Propagation error on the TP_Glu/Phe_ value based on 1*σ* on the *δ*^15^N measurement of amino acids in this study and 1*σ* on the *β* and TDF values reported in Chikaraishi et al. 2010.

4 Ref 2: Chikaraishi et al. (2010); Ref 3: Chikaraishi et al. (2011).
